# Aerosol Pollutants and Health: Role of Size and Chemical Composition

**DOI:** 10.1002/puh2.70134

**Published:** 2025-09-26

**Authors:** Samar Damiati, Buthaina A. AlMashrea, Nima Rabiei, Anju Prakasan Sujatha, Dana Khdr Sabir, Mohamad Alhosani, Rimantas Kodzius

**Affiliations:** ^1^ Chemistry Department, College of Sciences University of Sharjah Sharjah UAE; ^2^ Chemical Analysis Laboratories Section, Dubai Central Laboratory Department Dubai Municipality Dubai UAE; ^3^ Engineering and Natural Sciences International University of Sarajevo Sarajevo Bosnia and Herzegovina; ^4^ Research & Development, Bee'ah – Sharjah Environment Company Sharjah UAE; ^5^ Department of Pharmaceutical Chemistry, College of Science Charmo University Chamchamal Kurdistan Region Iraq; ^6^ Vilniaus kolegija / Higher Education Institution Vilnius Lithuania; ^7^ Health Care Faculty, Siauliu valstybine kolegija / Higher Education Institution Siauliai Lithuania; ^8^ Faculty of Medicine Ludwig Maximilian University of Munich Munich Germany

**Keywords:** aerosols | organ‐on‐a‐chip (OoC) | particulate matter (PM_10_ and PM_2.5_) | toxicity | ultrafine particles

## Abstract

Air, an essential environmental component that surrounds us everywhere and is easily inhaled, is no longer the same as it was hundreds or thousands of years ago. Today, it carries a wide range of suspended particles and pollutants originating from both natural phenomena—such as dust storms and volcanic activity—and anthropogenic sources like vehicle emissions and various human activities. Particulate matter in the air is often classified by size, including PM_10_, PM_2.5_, and ultrafine particles, but particle size alone does not determine their behavior or impact. Other important characteristics, including surface area, chemical and biological composition, aspect ratio, and electric charge, also play a critical role in how these particles interact with living systems and the environment. These pollutants, especially with long‐term exposure, pose serious threats to human health, ecosystems, and the environment. Recent advances in microfluidic technologies have enabled more precise assessment of air pollutant toxicity and exposure effects on human tissues. Among these, organ‐on‐a‐chip (OoC) devices are particularly valuable for measuring the toxicity of pollutants on various human tissues and for quantifying the pollutant. In this article, in addition to extensively reviewing the fundamentals and recent developments in the field of aerosol pollutants, we present a simple mathematical explanation demonstrating how surface area plays a significant role in the interaction of biological molecules or chemicals with surfaces. A promising approach involves collecting data from ground‐based and satellite monitoring and integrating it into predictive models, which may help in understanding and identifying potential air pollution sources and mitigating exposure.

## Graphical abstract

GRAPHICAL ABSTRACT

This review article focuses on air pollution arising from natural and human sources, with particle properties influencing health and environmental effects. Modern technology, organ‐on‐a‐chip (OoC), provides promising tools for assessing these impacts.

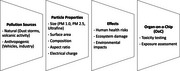

## 1 Introduction

Quality of life is a crucial aspect in the 21st century, and air pollution is a significant factor contributing to various challenges. It not only impacts human health but also poses a threat to the whole ecosystem, with its effects becoming increasingly evident over time. The air we breathe is reducing its quality due to the severe damage inflicted on ecosystems, making air pollution one of the top risk factors to human well‐being [1]. Furthermore, air pollution is transboundary pollution, considered one of the deadliest environmental hazards, resulting in an estimated $8.1 trillion in global health costs related to exposure, according to World Health Organization (WHO) reports (2025). The reports of WHO [2] and World Bank [3] clearly show that the air‐related risk causes 7 million premature deaths every year and is equivalent to the number of people that have died from COVID‐19 since March 2020 [2, 3]. According to the WHO, recent reports show that 43% of the deaths are due to chronic obstructive pulmonary disease; among them, 95% of deaths are caused in low‐ and middle‐income countries. In individual countries, the economic burden of pollution associated with premature mortality and morbidity is also significant, equivalent to 5%–14% of countries’ GDPs [3]. The study of Organization for Economic Cooperation and Development (OECD) projections shows that the expected increase of global healthcare costs related to air pollution is from $21 billion in 2015 to $176 billion in 2060 [4].

Air pollutants, typically a mixture of gaseous substances, particulate matter (PM), and water vapor, originate from both natural and anthropogenic sources. Natural sources include forest fires, volcanic eruptions, wind‐related abrasions, sandstorms, marshy ecosystems, and the decomposition of organic substances [5, 6]. Anthropogenic sources include combustion of fossil fuels, industrial activities, agricultural practices, landfill emissions, deforestation, land use changes, improper waste disposal, and sewage treatment plants, release the air pollutants into the air environment [7, 8, 9, 10]. Impacts of these pollutants can cause serious illness in human beings and degradation of the biotic ecosystems and enhanced climate change. The engine combustion process produces various gases and particles, posing health damage. Petroleum and oil contain various by‐products, including complex sulfur‐containing molecules. When these fuels are burned, sulfur oxides (SO*_x_*), particularly sulfur dioxide (SO_2_), are released into the atmosphere. Presence of catalysts such as nitrogen dioxide (NO_2_) activates sulfur dioxide to undergo further oxidation and form hazardous sulfuric acid (H_2_SO_4_). Additionally, the incomplete combustion of organic matter leads to the emission of carbon monoxide (CO), a particularly dangerous pollutant. Acute CO poisoning has serious health risks, as the gas readily enters the lungs and binds with high affinity to hemoglobin and mitochondrial cytochrome oxidase, thereby impairing the body's oxygen transport and utilization. CO exposure can also trigger lipid peroxidation in brain tissues, especially affecting unsaturated fatty acids, which may result in necrosis of white matter. Chronic exposure to low levels of CO is also harmful, potentially causing persistent neurological issues such as memory loss, depression, and headaches, and exacerbating cardiovascular conditions. Although symptoms often resolve upon removal of the CO source, this is not always the case in severe, acute poisoning events [11, 12].

Numerous efforts are going on worldwide to reduce air pollution, focusing on implementing regulations, adopting cleaner technologies, and preserving natural ecosystems to mitigate emissions from both human‐made and natural sources. Air composition is constantly altered by the introduction or removal of extraneous substances resulting from natural processes or human activities. The air composition changes: It seriously affects the troposphere, hydrosphere, and biosphere. The particle size of the air environment ranges from several millimeters to micrometers and even up to nanometers. Ambient air pollution consists of gaseous components and PM; the former includes CO, SO*_x_*, ozone (O_3_), volatile organic compounds (VOCs), and nitrogen dioxide (NO_2_), and the latter consists of particles in the air environment. Airborne particles are categorized by size, such as PM_10_, PM_2.5_, ultrafine particles (UFPs) (aerodynamic diameter of <0.1 µm), and nanoparticles. UFPs and nanoparticles are significant contributors to human health issues. Besides particle size, various other factors influence the air environment and the behavior of these particles [13], impacting biodiversity and overall health. Accurate identification of air pollutants is crucial for evaluating the potential risks posed by atmospheric contaminants, including particulates, nanoparticles, bioaerosols, and certain chemicals, especially in relation to pulmonary exposure and human health [14].

The composition of the air environment significantly affects the physical and chemical characteristics of airborne particles by processes such as condensation, adsorption of gaseous species, and heterogeneous chemical reactions, which can alter particle size, morphology, hygroscopicity, and toxicity [15, 16]. Air particles pose different characteristics, such as surface area, chemical and biological composition, aspect ratio, and charge. UFPs are emerging as one of the dominant and abundant PMs. Human exposure to these particles has increased dramatically, especially in the indoor environment, as most of the population spends 80%–90% of their time in the indoor environment. The small size of UFPs allows them to penetrate deep into the respiratory tract, causing effects from mild respiratory issues to cardiovascular and respiratory mortality, lung cancer, neurological diseases, and mutagenic or carcinogenic impacts [17, 18, 19]. Unlike PM_10_ and PM_2.5_, UFPs remain less studied, yet they contain organic compounds, toxic elements, nitrates, sulfates, and trace metals. Their fine structure enables transport through the respiratory system into tissues via the bloodstream, where they can generate reactive oxygen species (ROS), leading to genotoxicity, neurotoxicity, cardiovascular disease, and cancer [20, 21, 22].

The organ‐on‐a‐chip (OoC) microfluidic chips are small, lab‐grown models that replicate the structure and function of human organs using microfluidic technology. These chips allow researchers to simulate the behavior of human tissues and organs at a cellular level [23, 24]. It helps to detect the pollutant toxicity measurements on various body tissues.

## 2 Aerosols

A collection of microscopic solid and/or liquid particles suspended in the air atmosphere is called aerosols. Their behavior and size are determined by the physical and chemical properties of the surrounding air, such as temperature, humidity, and composition, together with particle interactions and atmospheric processes [25, 26]. The PM is mixed with toxic chemicals or biological particles, and its composition may vary depending on its source of origin, such as land or water. Contrary to their size, they play an important role in the earth's climate formation by cloud setting, condensation, precipitation, and blocking the radiation from entering the earth's surface [27].

Aerosols can originate from both natural sources like wind‐driven dust, sea spray, and volcanic eruptions, as well as from human activities such as fuel combustion and urban processes [28]. In densely populated areas, aerosols primarily consist of PM, with gaseous components generated from various emission sources. PM, especially when small enough, can be inhaled deeply, leading to serious health consequences, including premature death, heart attacks, and negative effects on pregnancy outcomes [29]. Dust, haze, smoke, pollutants, fumes, mist, and fog are all forms of aerosols, typically with diameters under 1 µm. Due to their small size, PM_2.5_, PM_1_, UFPs, and nanoparticles can penetrate the lungs, respiratory system, and other internal organs, resulting in harmful health effects.

Dust and sandstorms are major natural sources of PM_10_ and PM_2.5_ in arid regions and merit more detailed consideration. These events elevate particulate levels substantially, as shown by ground‐based monitoring and satellite‐derived aerosol data combined with models such as ERA5 and HYSPLIT [30]. Their particles, composed largely of silicates, carbonates, and oxides with trace metals and organics, pose toxicological risks. Epidemiological studies link dust exposure to increased hospital admissions and mortality from respiratory and cardiovascular diseases, particularly in individuals with asthma and COPD [31, 32].

### 2.1 Role of Carbon Monoxide and Other Gaseous Pollutants in Aerosols

Airborne particles remain suspended depending on size, temperature, and humidity, with particles smaller than 10 µm able to persist for weeks [33]. Coal combustion releases H_2_O_2_ and sulfur compounds, particularly SO_2_, which can undergo atmospheric oxidation in the presence of NO_2_ to form H_2_SO_4_, a key precursor of sulfate aerosols:

$$SO_{2}\left(g\right)+NO_{2}\left(g\right)+H_{2}O_{2}\left(\mathrm{aq}\right)\to H_{2}SO_{4}\left(\mathrm{aq}\right)\to \mathrm{sulfate}\mathrm{aerosols}$$

Combustion of organic matter also emits CO, which binds preferentially to hemoglobin (Hb), myoglobin, and mitochondrial cytochrome oxidase, impairing oxygen transport and utilization:

Hb+CO→HbCO

This can cause brain lipid peroxidation and white matter necrosis [34, 35]. Chronic low‐level CO exposure can lead to memory loss, depression, headaches, and cardiovascular issues, though symptoms usually resolve once the source is removed unless acute poisoning has occurred [36].

### 2.2 Role of Metals/Salts in Aerosols

Nano‐sized particles are produced both indoors and outdoors during industrial (e.g., TiO_2_ production) or mechanical processes (e.g., sanding, drilling). Workers should take precautions against exposure. Fossil fuel and biomass combustion, as well as motor engine exhaust, are major anthropogenic sources of aerosols, forming organ sulfates in heavily polluted regions [37]. Urban aerosols typically contain 20%–50% carbonaceous material, including organic carbon (OC), elemental/black carbon (EC/BC), and carbonate carbon (CC) [38, 39]. Natural sources include sea spray, dust storms, and volcanic eruptions, which release ions (Na^+^, Mg^2+^, Ca^2+^, K^+^, SO_4_^2−^) and gases (H_2_S, HCl, H_2_SO_4_) that contribute to aerosol formation, transport pollutants, and drive chemical reactions in the atmosphere [40, 41, 42].

### 2.3 Bioaerosols

Bioaerosols are defined as airborne particles of biological origin, including microorganisms and their by‐products. The role of aerosols in the transmission of SARS‐CoV‐2 is increasingly relevant in this decade and is the discussed topic since the spread of the COVID‐19 pandemic. The bio‐particles in the air environment range in different sizes, diversity, and composition and are of diverse taxonomic origins that include algae, oomycetes, fungi, bacteria, archaea, viruses, plant pollen, spores, and organic compounds such as toxins and proteins and are transported as particles associated with viruses and bacteria or individual entities such as pollens and fungal spores. The particle diameter of bioaerosols is in the range of 10–100 mm, which are ejected into the atmosphere from the biosphere, and epidemiological issues are increasing day by day due to the impacts of bioaerosols [43, 44].

## 3 Classification Based on the Size of the PM

**FIGURE 1:**
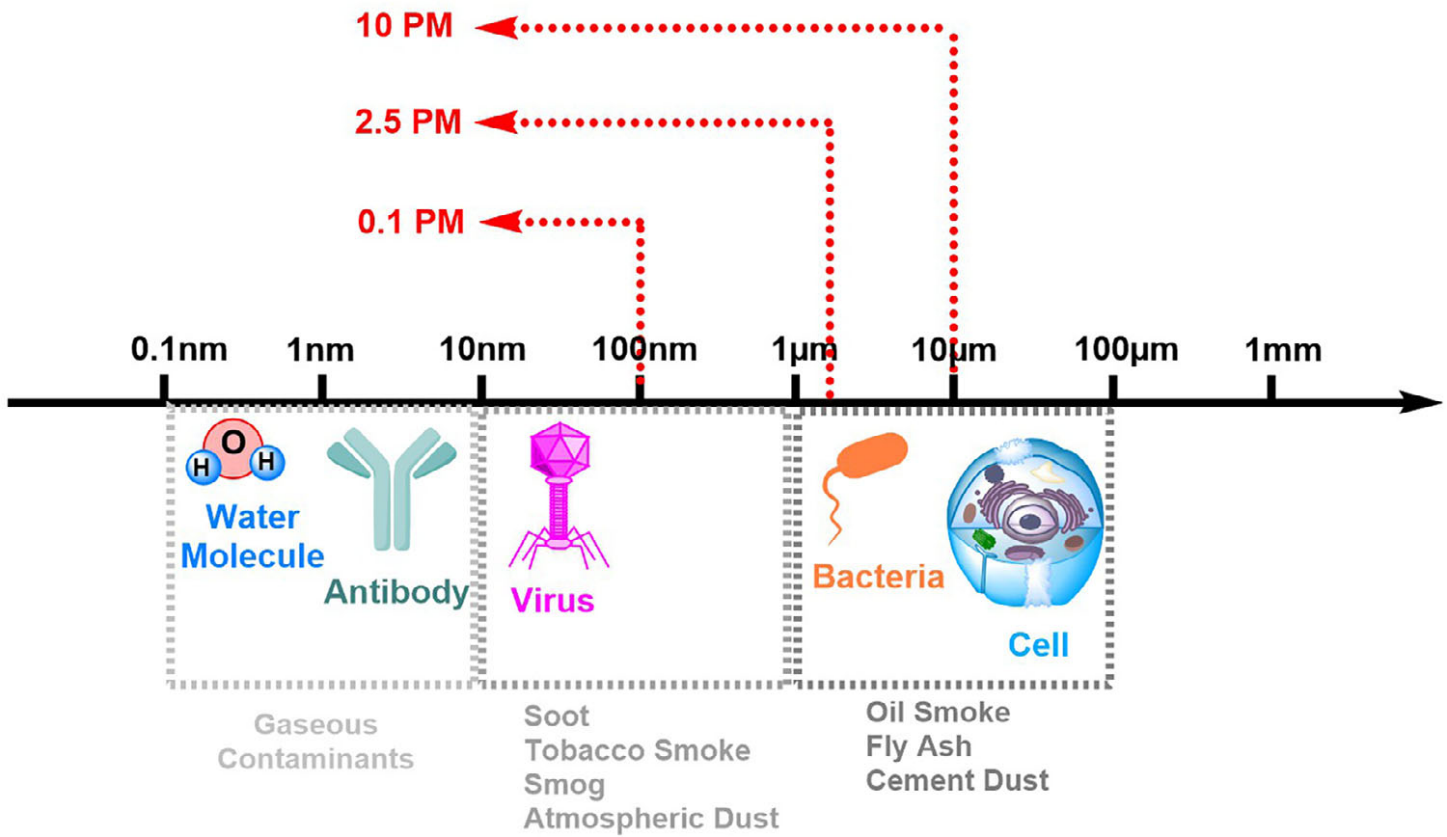
A size comparison of different contaminants, including gaseous pollutants, soot, smoke, and dust particles with various chemical and biological molecules and cells. PM_0.1_, PM_2.5_, and PM_10_ indicate the median particle diameter for each particulate matter category.

All microscopic liquid or solid matter suspended in the atmosphere is referred to as PM (Figure 1). The size of PM is critical, and thus, air particle distribution is classified on the basis of the mass and volume distribution of the particles. Gaseous pollutants are generally smaller than viruses, with sizes typically between 0.1 and 10 nm. Particles, such as soot, tobacco smoke, and smog, are comparable in size to small viruses or fine atmospheric dust, ranging from 0.01 to 1 µm. Substances like fly ash, oil smoke, and cement dust fall within the size range of certain allergens (like dust mites), as well as bacteria, mold spores, and pollen, typically between 1 and 100 µm.

Particles, such as dust, dirt, and soot, are large and dark enough to be seen with the naked eye, and they settle down fast. Suspended particles are so small that they aren't settled normally and can only be detected by using an electron microscope or more sophisticated methods. Depending on their aerodynamic diameter, particles are classified as coarse (2.5–10 µm), fine (≤2.5 µm), or ultrafine (≤0.1 µm). Accordingly, PM_10_ refers to particles with aerodynamic diameters ≤10 µm, PM_2.5_ to those ≤2.5 µm, and PM_0.1_ to those ≤0.1 µm. PM_10_ is inhalable particles, with diameters that are generally 10 µm and smaller, which are mainly deposited in the tracheobronchial region, and PM_2.5_ are fine inhalable particles, with diameters that are generally 2.5 µm and smaller [45]. PM_2.5_ has a kinetic energy equivalent diameter of less than or equal to 2.5 µm in the atmosphere [46] and consists of tiny particles or droplets in the air that are smaller, that is, 30 times more than the width of a human hair, and easily enter the respiratory system when inhaled, traveling deep into the lungs and then to the bloodstream. PM less than <0.1 µm and particle‐bound polycyclic aromatic hydrocarbons (PAHs) from combustion of biomass, which penetrate the lung alveoli and enter into the bloodstream and directly affect the circulatory system, are suspended in the air for a long time [47, 48].

### 3.1 Airborne Pollutants

Airborne pollutants can also be classified on the basis of particle size and its impact on human health (Table 1). High‐volume air samplers, capable of processing over 1500 m^3^ of air in 24 h, are used to measure total suspended particulates (TSPs), representing the full range of particles present in the atmosphere. Larger particles are often referred to as Suspended PM (SPM), with definitions varying depending on the measurement equipment. For instance, DustWatch monitors define SPM as particles up to 100 µm in diameter. Within SPM, smaller particles are classified as respirable suspended PM (RSPM), also known as respirable suspended particulates (RSP). RSPM is more hazardous to health, and the RSPM‐to‐SPM ratio is often a more relevant health indicator than SPM alone.

**TABLE 1:** Characteristics of particulate matter (PM) and their impact on human health.

PM feature	Influence on health
Particle size	Smaller particles can penetrate more easily and deeply into the body's organs
Particle shape	Irregular or elongated particles, such as asbestos, tend to cause greater damage, particularly to lung tissue
Surface charge	Negatively charged nanoparticles may more easily cross the gastrointestinal mucus barrier compared to positively charged ones
Collective behavior of small particles	The health effects of the combined presence of small and large particles are not fully understood but may be cumulative
Chemical composition	Certain components, such as lead and polycyclic aromatic hydrocarbons, are highly toxic and potentially carcinogenic
Biologic carrier	Particles that carry protein structures resembling allergens can trigger immune responses

### 3.2 PM_10_ (PM < 10 µm)

PM_10_ is primarily generated by industrial activities, combustion processes, and vehicles. Some sources define PM_10_ as particles with a diameter of 10 µm or smaller [49]. However, some researchers assume this definition is not entirely precise, as the measurement equipment typically determines the median particle diameter, and it can be affected by the humidity and temperature variations. Hence, PM_10_ refers to the concentration of particles with a median size of 10 µm. This means that 50% of the particles will have diameters smaller than 10 µm, whereas the other 50% will be larger than 10 µm.

Identification of PM_10_ mass concentration is important to meet and ensure regulatory compliance. PM regulations are based on reference methods, which use gravimetric analysis of filters at a specific temperature and RH [50]. In the gravimetric analysis, calibrating low‐cost sensors with reference methods is difficult because procedure is manual and generally yields low time resolution (24 h), and the filter‐based gravimetric takes considerably longer time. One of the real‐time measurement and continuous monitoring methods is the beta attenuation method (BAM) by using beta attenuation monitors, where a beam of beta radiation is passed through a filter where PM is collected, and the particles attenuate the radiation, and the amount of attenuation is proportional to the mass of the collected particles [51].

### 3.3 PM_2.5_ (PM < 2.5 µm)

Fine PM (PM_2.5_), which consists of particles with aerodynamic diameters smaller than 2.5 µm, is associated with several negative health effects [52, 53]. PM_10_ particles are produced by both combustion and non‐combustion processes, including industrial activities, motor vehicle engines, sea salt, windblown dust, and fires. In contrast, PM_2.5_ particles are primarily generated by combustion processes, such as fires, industrial boilers, solid fuel heaters, and motor vehicle engines. Research has shown that smaller particles, particularly those smaller than 2.5 µm in diameter, are more strongly linked to adverse health effects compared to PM_10_ particles [54]. Both PM_2.5_ and PM_10_ particles are invisible to the naked eye. For comparison, a particle measuring 2.5 or 10 µm in diameter is much smaller than a human hair, which is about 60 µm thick. Some publications define PM_2.5_ particles as only particles smaller than 2.5 µm, which is understandably oversimplified. Both short‐term and long‐term exposures to these particles are linked to increased mortality [55, 56, 57]. In addition to health effects, these invisible particles can harm the ambient environment, reduce visibility, and damage materials and structures [53, 58]. According to the International Standards Organization (ISO), the PM_2.5_ value refers to “particles that pass through a size‐selective inlet with a 50% efficiency cut‐off at an aerodynamic diameter of 2.5 µm” [47, 53]. PM_2.5_ particles are more challenging to measure compared to PM_10_. The PM_2.5_ fraction possesses particles with a median diameter of 2.5 µm, though it naturally contains both larger and smaller particles around that size.

The PM_2.5_ fraction differs from PM_10_ primarily in the size of the particles measured; however, some of the same equipment can be used for both but with different filters applied. However, measuring PM_2.5_ is more challenging because the total mass of PM_2.5_ particles is lower than that of larger particles. As a result, the coarse fraction must be excluded, requiring more precise measurement techniques. The principles used to measure the smaller PM_2.5_ particles are similar to those for PM_10_, but the reference methods for PM_2.5_ do not provide real‐time data. There are commercially available instruments designed for PM_2.5_ measurements, such as the Partisol manual gravimetric samplers (e.g., Partisol 2025) [59, 60]. These gravimetric samplers use a sequential filter sampling system, allowing for several fixed 24‐h period samples and requiring filter cassette replacements every 2 weeks.

### 3.4 Ultrafine Particles

The most harmful particles to human health are in the nanometer size range, capable of penetrating cell membranes and migrating to internal organs such as the brain, as well as affecting the cardiovascular system [61]. UFP, typically defined as particles smaller than 100 nm (<0.1 µm) in diameter, are also referred to as PM_0.1_. Because UFPs contribute minimally to overall particle mass, their measurement focuses on particle number rather than mass concentration. Traditional light scattering methods are ineffective at detecting these tiny particles, so instruments like the condensation particle counter (CPC) are used instead [62, 63]. In CPCs, air is passed through a chamber saturated with alcohol vapor, which condenses on the UFPs, enlarging them so they can be counted optically. CPCs can measure particles ranging from 3 to 2000 nm. Another method, the scanning mobility particle sizer [64, 65], classifies particles based on electrical mobility and measures concentrations within the 11–450 nm range. As previously mentioned, smaller and lighter particles—particularly those under 10 µm—remain suspended in the air longer, whereas those under 1 nm can persist for weeks. For example, diesel PM (DPM), which is typically smaller than 100 nm, is emitted from diesel engines and is found in highest concentrations near sources such as highways or urban areas [66, 67]. These particles are generally removed from the atmosphere through precipitation, such as rainfall. The PM_0.1_ particles are less than 100 nm. Specifically, it has been recognized that particles with a size smaller than 300 nm are considered atmospheric nanoparticles [68, 69, 70].

## 4 Parameters Shaping the Particle Effects

Although particle size in the atmosphere significantly influences cell and tissue health—and by extension, overall human health—other factors are equally important. The surface area of a particle is closely linked to its size, with smaller particles, such as nanoparticles, exhibiting collective behaviors often described by quantum physics. The chemical composition of a particle determines its reactivity and interactions with both other substances and biological cells. Additionally, biological materials like DNA and proteins attached to particles can affect cellular functions, particularly by influencing the immune system.

### 4.1 Particle Surface Area

The surface area of airborne PM refers to the total surface exposed on particles, which allows for interactions with gases, biological cells, and other substances in the atmosphere [71]. This surface area plays a crucial role in how particles engage with environmental and biological systems. Larger particles tend to exhibit greater chemical reactivity compared to smaller ones, influencing atmospheric processes such as oxidation and reduction and contributing to cloud formation. PM with a diameter of 10 µm or less can have substantial surface areas and may be inhaled into the lungs. Particles that are 2.5 µm or smaller have an even greater surface area‐to‐volume ratio, making them more reactive and potentially more dangerous. UFPs, which are smaller than 0.1 µm, have the highest surface area per unit mass and pose significant health risks, as they can penetrate deep into the lungs or even enter the bloodstream [72, 73, 74].

Numerous studies indicate that the health risks associated with airborne particles are closely linked to their size. Smaller particles can penetrate more deeply into the lungs and even reach the cardiovascular system. Once they enter the body, these particles can accumulate, resulting in various organ damage. Standard measurement techniques typically investigate the total mass of specific particle size fractions, such as PM_10_ or PM_2.5_. Although particle counts can be estimated from these mass measurements, total mass alone is not an accurate indicator of health risk. For instance, a single particle with a diameter of 10 µm has the same mass as approximately 1 million particles measuring 100 nm each. It is clear that a single large particle poses less of a threat than a vast number of smaller ones, which are more likely to reach the alveoli and cause harm.

**FIGURE 2:**
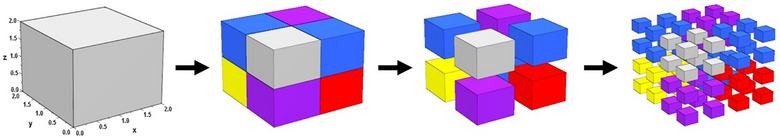
Dividing the cube into eight smaller sub‐cubes increases its surface area from 24 to 48 m^2^. Further division into 64 smaller sub‐cubes increases the surface area to 96 m^2^.

In this article, we present a straightforward mathematical explanation of how a cube can be divided into eight smaller cubes, illustrating the resulting increase in surface area (see Figure 2). Notably, the total volume remains unchanged. We begin by considering a cube is given: B={(x,y,z)|0≤x≤l,0≤y≤l,0≤z≤l}=[0,l]×[0,l]×[0,l]. Subsequently, the surface area of the cube is given by $s=6l^{2}$. When the cube is divided into smaller sub‐cubes, the total surface area increases. This is achieved by subdividing each of intervals [0,l] into n sub‐intervals $\left[l_{i-1},l_{i}\right]$ of equal width $\Delta l=\frac{l}{n}$. The planes through the endpoints of these sub‐intervals parallel to the coordinate planes divide the box B into $n^{3}$ sub‐boxes $B_{ijk}=\left[l_{i-1},l_{i}\right]\times \left[l_{j-1},l_{j}\right]\times \left[l_{k-1},l_{k}\right]$. Each sub‐box has surface area $\Delta s=6\;\Delta l^{2}=6\frac{l^{2}}{n^{2}}$. Then the sum $T_{s}=\sum_{i=1}^{n}\sum_{j=1}^{n}\sum_{k=1}^{n}\Delta s=n^{3}\Delta s=n6l^{2}=ns$ was formed to obtain the total surface area, where s is the surface area of the cube B. Thus, after dividing the cube into $n^{3}$ smaller sub‐boxes, the total surface area becomes *n* times greater than that of the original cube B, that is, $\frac{T_{s}}{s}=n.$ For example, in Figure 2, l=2 and the box is divided into $2^{3}=8$ sub‐boxes.

The original box has a surface area of 24 m^2^. After the first division, the total surface area doubles to 48 m^2^ (i.e., $48=2\times 24\;m^{2}$). A subsequent division of each resulting sub‐cube into eight smaller cubes increases the total surface area to 96 m^2^.

As mentioned earlier, particle surface area is a critical factor in determining health impacts. For equal mass fractions, smaller particles have a significantly larger total surface area due to their higher number concentration compared to larger particles (see Figure 2). Because particle interactions with human cells and intracellular structures occur via their surfaces, this increased surface area enhances their potential to disrupt vital enzymatic processes [75]. However, directly measuring particle surface area is challenging. One approach involves attaching heavy labeled atoms (e.g., ^211^Pb) to the particles. These aerosols are collected on a filter, and the resulting signal is detected using an alpha counter (such as an epiphaniometer). The measured signal reflects the transferred mass, which is proportional to the particle surface area [76]. Another method estimates surface area based on particle number size distributions. By assuming a particle shape and geometry, these distributions can be converted into surface area values. Typically, a scanning mobility particle sizer is used to measure smaller particles (ranging from 2.5 to 1000 nm) [64], whereas an aerodynamic particle sizer is used for larger particles in the 0.5–20 µm range [77, 78].

Surface area plays a critical role in interactions between biological molecules or chemicals and material surfaces. For example, a large surface area can adsorb proteins such as polymerases, rendering them inactive and unavailable for biological reactions [75]. This work demonstrates how biological molecules, such as enzymes, can be inhibited upon contact with microfluidic material surfaces. In contrast, for environmental pollutants, a large surface area can have harmful effects, especially on lung cells, by promoting toxic interactions.

Accurate data on particle number, morphology, size, and surface area are essential for understanding health risks. It is well established that PM_10_ and PM_2.5_ pose greater health risks than larger particles up to 100 µm in size. Substantial evidence links PM_2.5_ and PM_10_ exposure to increased hospital admissions and premature deaths among vulnerable populations, particularly the elderly and those with pre‐existing conditions. PM significantly contributes to all‐cause mortality, especially cardiopulmonary‐related deaths. Even short‐term exposure such as during dust storms or pollution episodes can worsen asthma symptoms and reduce overall activity levels. A review by Schulze et al. [79] summarizes the health impacts of various pollutant particles. For example, modern diesel engines emit a high number of DPM particles, often around 100 nm (0.1 µm) in size. These soot particles frequently carry carcinogens, such as benzopyrenes, adsorbed on their surfaces. Although particulate filters are being developed to mitigate these emissions, their effectiveness is limited in regions with weak regulatory oversight—such as some developing countries—where vehicles may lack such filters. It is also important to note that emission standards for diesel engines often focus on particle mass, which may not fully reflect the associated health risks.

### 4.2 Chemical Composition of PM

Understanding the chemical composition of airborne particles is crucial for identifying pollution sources and estimating particle distribution. Various studies have aimed to determine particle composition. For example, a 2008 study analyzing collected PM_2.5_ particles revealed that three major components—OC, nitrate (NO_3_^−^), and sulfate (SO_4_^2−^)—constituted a significant portion of the total mass [80]. Both primary emissions (e.g., road traffic) and domestic combustion (e.g., oil and solid fuels) contribute to the presence of EC and organics. Research has established a strong link between vehicle emissions and elevated BC concentrations. BC refers to the sooty material released from engines and other fossil fuel combustion sources. Hourly monitoring has shown that BC levels peak in the evenings, largely due to the use of solid and liquid fuels for residential heating [81].

Non‐exhaust traffic emissions, such as road surface abrasion and tire or brake wear, also contribute significantly to PM_2.5_ levels. These emissions increase with traffic volume and often include iron‐rich dust. Measuring iron is important for tracing non‐exhaust emissions, especially from vehicles. Even electric vehicles, though considered “clean,” still contribute through tire and brake wear. Other sources such as the construction industry, including demolition and handling of minerals and cement, as well as wind‐blown soil, release particles rich in calcium (Ca^2+^). Sodium chloride (NaCl) commonly originates from sea salt.

Elevated NO_3_^−^ levels have been observed during some PM_2.5_ pollution episodes [82, 83]. These findings highlight the need for comprehensive monitoring of both air quality and particle chemistry across urban and rural settings. A recommended minimum set of chemical species for routine measurement includes EC, OC, Na^+^, K^+^, Ca^2+^, Mg^2+^, Cl^−^, NH_4_^+^, NO_3_^−^, SO_4_^2−^, and Fe. Interestingly, the chemical pairing of nitrate and sulfate with ammonium suggests the presence of neutral salts, such as NH_4_NO_3_ and (NH_4_)_2_SO_4_. These compounds result from complex interactions between NH_3_, NO*_x_*, and SO_2_. Although ammonium sulfate is stable under atmospheric conditions, ammonium nitrate is thermally unstable and may decompose back into HNO_3_ and gaseous NH_3_ depending on the atmospheric conditions. In addition to primary emissions, road traffic contributes to secondary PM through oxidation of NO*_x_* emissions. A significant portion of PM found in the atmosphere forms secondarily from precursor gases such as ammonia, NO*_x_*, and SO_2_.

Lead (Pb) monitoring since 1985 has significantly contributed to our understanding of its health risks, eventually leading to the ban of leaded petrol in 2000 in many countries. However, approximately 18 nations, including Algeria, Yemen, and Iraq, still use leaded fuel due to short‐term economic interests [84, 85]. Lead exposure, especially in children, is associated with neurotoxic effects such as reduced IQ and antisocial behavior.

In some countries, such as the United Kingdom, metal concentrations in PM_10_ particles are routinely measured. Samples are collected using cellulose filters in low‐ or high‐volume air samplers. The filters are then dissolved in a mixture of HNO_3_ and H_2_O_2_, followed by analysis via inductively coupled plasma mass spectrometry (ICP‐MS). This sensitive method is particularly suited for trace element detection, including Ni, As, Cd, and Pb.

PAHs are another group of hazardous chemicals found in PM. These lipophilic, nonpolar compounds are known carcinogens and may also contribute to cardiovascular issues and fetal development disorders. PAHs are typically monitored in PM_10_ and PM_2.5_ particles. Advanced methods allow their detection in even finer fractions. Samples are collected using polyurethane foam plugs or filters and extracted with dichloromethane. Gas chromatography–mass spectrometry (GC–MS) is then used to quantify up to 32 different PAHs, including acenaphthene, anthracene, benzanthracene, benzofluoranthene, benzoperylene, benzopyrene, chrysene, fluorene, phenanthrene, and pyrene [86]. Table 2 summarizes the main findings of the reported studies.

**TABLE 2:** Chemical composition of particulate matter (PM) and sources.

Component	Typical source	Notes/Health implications	Refs.
Organic carbon (OC)	Vehicle emissions, domestic combustion	Contributes to PM mass; associated with oxidative stress	[80]
Elemental/Black carbon (EC/BC)	Fossil fuel combustion, diesel engines	Peaks in evenings due to residential heating; linked to cardiovascular and respiratory issues	[81]
Nitrate (NO_3_^−^)	Secondary formation from NO*_x_*	Forms ammonium nitrate; elevated during pollution episodes	[82, 83]
Sulfate (SO_4_^2−^)	Secondary formation from SO_2_	Forms ammonium sulfate; stable under atmospheric conditions	[82, 83]
Ammonium (NH_4_^+^)	Secondary formation	Combines with NO_3_^−^ and SO_4_^2−^ to form neutral salts	[82, 83]
Calcium (Ca^2+^)	Construction, demolition, wind‐blown soil	Traces mineral dust	[82, 83]
Sodium/Potassium (Na^+^, K^+^)	Sea spray, combustion	Traces of marine and combustion sources	[82, 83]
Magnesium (Mg^2+^)	Sea spray, soil dust	Natural source	[82, 83]
Chloride (Cl^−^)	Sea salt, industrial emissions	Influences particle reactivity	[82, 83]
Iron (Fe)	Non‐exhaust traffic emissions (tire/brake wear)	Traces non‐exhaust traffic PM; even EVs contribute	[82, 83]
Lead (Pb)	Historical leaded petrol, industrial emissions	Neurotoxic, especially in children; banned in many countries	[84, 85]
Polycyclic aromatic hydrocarbons (PAHs)	Combustion	Carcinogenic; may affect cardiovascular health and fetal development	[86]

The particle surface can influence the composition of airborne particles. The presence of coating or absorbed materials (e.g., water, organic compounds) on the particle can influence the surface area measurements and the reactivity. Research done by Alves et al. showcased that the chemical composition or heterogeneity of PM‐bound substances is important to determine its toxicity [87]. Combustion‐derived primary particulate components appear to play a key role in triggering inflammation and oxidative stress, which likely account for many of the observed negative health effects. The study done on an industrialized area where PM_2.5_ air monitoring campaigns were conducted in 3 years showed that the difference in the chemical composition has resulted in negative impacts on the human health [88].

### 4.3 Shape and Morphology of Particle

Particle shape is also a critical factor. Asbestos, for example, is composed of long, microscopic fibrils ranging from 0.1 to 10 µm in length, often exhibiting an aspect ratio of around 1:20. When inhaled, these sharp, needle‐like particles can penetrate deep into the lungs, causing significant damage. Generally, the greater the aspect ratio, the higher the toxicity of the asbestos particles [89]. In the context of nanomaterials, particles are typically categorized as one‐dimensional, two‐dimensional (2D), or three‐dimensional (3D). Asbestos fibers fall into the category of 2D nanoparticles.

Some particles are in irregular shapes (e.g., soot or dust) and have more surface area due to this irregularity than spherical particles such as water droplets and vapors. Surface roughness also contributes to the total surface area. Morphology is one of the important properties with particle size and coating when considering the particle characteristics in the air environment [90]. For the estimation of surface area of a particle (SA) and mass, additional information such as effective density, primary particle size, and morphology is required [91, 92]. The effective density (*ρ*_eff_) as defined from simultaneous tandem measurements of mobility size (*d_m_*) and mass (*m*), can be measured with the differential mobility analyzer–aerosol particle mass analyzer (DMA–APM), further described by McMurry [93]:

$$\rho_{\mathrm{eff}}\left(d_{m}\right)=\frac{6m}{\Pi {d^{3}}_{m}}$$

### 4.4 Agglomeration

Airborne particles can collide and stick together, forming larger agglomerates that reduce the effective surface area compared to individual particles. Soot particles, mainly generated from diesel engines and diffusion flame soot generators under varying conditions, exhibit a specific surface area ranging from 100 to 260 m^2^/g. Their size distribution, classified according to equivalent mobility sizes, influences agglomerate formation, transport, deposition in the human respiratory tract, and mass transfer during condensational growth in ambient air [92]. Engineered nanoparticles in the air add further complexity, existing in diverse shapes, sizes, chemical compositions, and degrees of agglomeration, which may impact their potential health effects [94]. Agglomeration is influenced by particle properties and environmental factors such as temperature and humidity, highlighting its critical role in aerosol behavior and human exposure.

### 4.5 Particle Collective Behavior

In addition to the surface area and chemical composition of PM, there are many other important factors, both known and unknown, that need to be considered. Smaller particles, especially when present in large quantities, follow the laws of particle physics, which differ from those governing bulk materials [95]. Research has confirmed that air is a complex mixture that not only contains gases like CO, NO*_x_*, and SO_2_ but also suspended PM, which includes particles in the 30‐ to 2.5‐µm range (PM_30_, PM_10_, and PM_2.5_), as well as UFPs smaller than 0.1 µm, including nanoparticles [96]. These particles have distinct chemical compositions, origins, and health implications. Nano‐sized particles often display collective behaviors, and this group is sometimes referred to as nanoparticulate matter [97]. Nanomaterials also differ from bulk materials due to their unique surface and quantum effects [98]. The surface area of nanoparticles increases drastically, causing surface atoms to experience a different environment compared to those in the interior. This size effect is particularly significant for nanoparticles smaller than approximately 5–50 nm [99]. For instance, a nanoparticle with a size of 1 or 100 nm contains several hundred to thousands of atoms. Moreover, it has been observed that particles smaller than 0.1 µm (PM_0.1_) can aggregate into larger particles, around the size of PM_2.5_ [100].

### 4.6 Particle Charge

When artificially producing various types of nanoparticles in the laboratory, it is important to consider their composition (whether made from a single material or multiple materials), as well as their magnetic and electromagnetic properties. These properties can impact how nanoparticles cluster (agglomerate), with the resulting particle clusters behaving like larger particles. Some studies have shown that negatively charged latex nanoparticles diffuse more quickly through the mucus layer of the gastrointestinal tract, whereas positively charged particles tend to get trapped in the negatively charged mucus [99, 101].

### 4.7 Source of Emission

The origin of particles influences their characteristics and effects. The formation of UFPs mainly results from the combustion of forest fires, coal, hydrocarbons, and biomass burning (e.g., agricultural burning, forest fires, and waste disposal), as well as vehicular emissions [102, 103, 104]. Vehicular emissions often produce fine particles rich in organic compounds and metals. Industrial emissions can generate particles containing heavy metals, sulfates, and other harmful substances. Biomass burning and wildfires release fine organic particles, including BC, which have significant health and climate impacts. Agricultural activities contribute to the release of ammonia and dust particles, which may react in the atmosphere to form secondary PM, such as ammonium nitrate.

### 4.8 Environmental Factors

Weather conditions influence particle surface area and impact temperature, humidity, and wind patterns, which in turn affect how particles behave in the air. High humidity promotes particle growth through condensation, making them larger and more likely to be inhaled. Temperature inversions can trap pollutants near the ground, increasing particle concentrations in specific areas. Wind can either disperse particles over long distances or concentrate them in certain locations, influencing air quality and deposition patterns. Additionally, atmospheric conditions such as UV light, pressure systems, and other phenomena can drive chemical reactions that transform gases into PM, such as the formation of sulfate aerosols.

### 4.9 Biological and Genetic Information—Bearing Particles

Dust particles, including pollen grains from male seed plants, serve as carriers of genetic (DNA) and biological (e.g., proteins) information. When inhaled, pollen can trigger allergic reactions in the human immune system. The proteins on the surface of pollen are primarily responsible for these immune responses, which can lead to conditions like allergies or asthma. The immune system can cross‐react to a variety of allergens, which are the carriers of these immune‐triggering proteins. A protein with a similar structure, regardless of its source (e.g., fleas and cat fur), can potentially provoke an immune reaction [105, 106, 107, 108]. Efforts are being made to classify allergens and their associated proteins based on the DNA that encodes them. From the studies of Sompornrattanaphan et al., the biological plausibility of the etiologic association between PM and allergic sensitization is supported by experimental evidence indicating PM could enhance immunologic responses to allergens and induce inflammatory reactions in the airways [108].

## 5 Assessment of Pollutant Toxicity

Toxicity testing for health and environmental regulations must take into account nanoparticle size, surface reactivity, and their tendency to agglomerate. Many everyday products contain nano‐ and microparticles, including food additives, toothpaste, cosmetics, sunscreens, stain‐resistant fabrics, and tires. Although some nanoparticles such as TiO_2_ used in sunscreens are well studied and standardized, many newer or less‐characterized particles require thorough toxicity assessments before they are approved for use in consumer products. It is also crucial to consider not just human health impacts but also how these particles are distributed into the environment, for example, through tap water reaching rivers and oceans, where they may affect aquatic life. At present, there is no clear or universal correlation between a nanoparticle's classification, such as its dimensionality, morphology, composition, or state of agglomeration, and its health effects. Importantly, not all nanoparticles are harmful; some are considered non‐toxic, and others have valuable applications in healthcare [109, 110, 111].

### 5.1 Microfluidic Devices for the Detection and Assessment of Air Pollutants

Advancements in the field of OoC technology enable long‐term monitoring of mammalian cell growth. By introducing chemicals that mimic pollution sources, researchers can observe cellular responses and identify abnormalities. This approach allows for the assessment of toxicity and determination of effective dose levels. OoC offers a rapid, ethically sound alternative to animal testing and can be readily implemented in any laboratory equipped for mammalian cell culture [84, 112]. Many initiatives and efforts are bolstered globally to elevate air quality over management and a controlling system for monitoring different pollutant types by adopting state‐of‐the‐art systems and technology. One of these technologies is a microfluidic chip system. Microfluidic technology has two sides to the working path, which can be used either as lab‐on‐chip (LOC) for identifying and quantifying pollutants or as OoC for studying the toxicity effect of prolonged exposure to contaminants by simulating the behavior of human organs on a chip without involving animals’ organs [113, 114]. This technology opens new avenues of innovation in testing and integrates well with other techniques to achieve high efficiency and productivity in the application.

**FIGURE 3:**
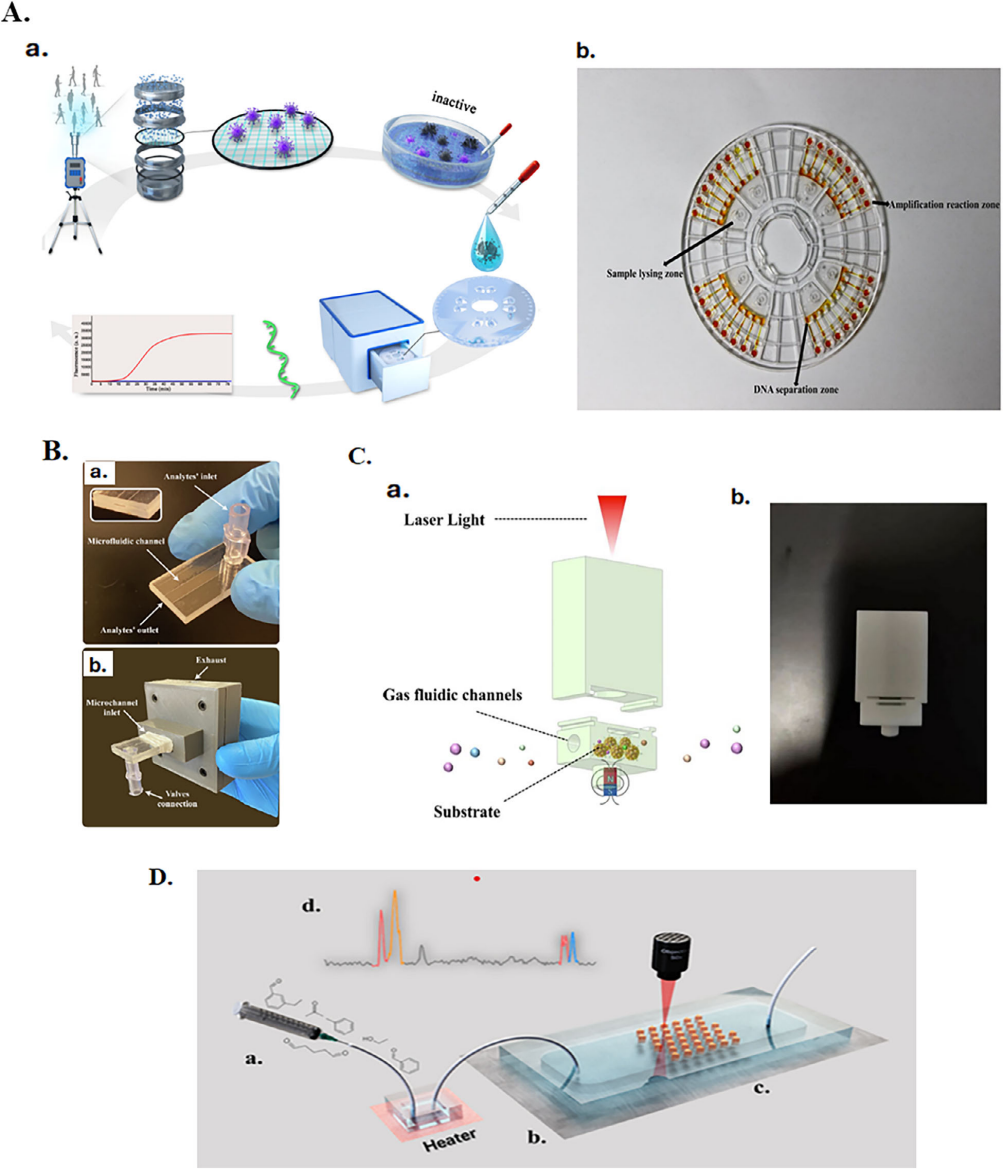
(A) a. Schematic diagram of the sampling and sensing aerosol SARS‐CoV‐2; b. the microfluidic chip filter membrane [115]. (B) a. Microfluidic chip component; b. connection of the chip with 3D housing [116]. (C) a. Schematic of the 3D‐printed model in connection with SERS detection; b. the real 3D‐printed device [117]. (D) The four steps of detection: a. The analyte mixture is pumped into a slot for heating; b. the gas flows into the SERS‐Array chip; c. the SERS probes capture the aldehydes; d. the label‐free SERS method detects multiplex aldehydes [118]. Source: (A) Reproduced from Analytical Chemistry [115] with permission from American Chemical Society (ACS). (B) Reproduced from Environmental Technology & Innovation [116] with permission from Elsevier. (C) Reproduced from Analytical Chemistry [117] with permission from American Chemical Society (ACS). (D) Reproduced from ACS AppliedMaterials & Interfaces [118] with permission from American Chemical Society (ACS).

The atmosphere is a complex system where microscopic solid particles and light gases can remain suspended, potentially accumulating over time and contributing to air pollution, with significant implications for environmental quality and human health. These pollutants can be in different forms, like viruses, particles (tiny or droplets), and gases like VOCs. In this section, different studies were presented to provide overviews of the applicability of using LOCs and OoCs in maintaining air quality. For instance, Xiong et al. [115] report research work on detecting SARS‐CoV‐2 on‐site in the air aerosol based on a microfluidic fluorescence system suitable for sampling and detection together, which elevates the device experiences and use for on‐site. His system has the advantages of rapid detection within 15 min, high sensitivity with a detection limit of 10 copies/µL, and a good precision of <5.0% compared to the traditional method of testing using reverse transcription polymerase chain reaction (RT‐PCR). The working process of this device is collecting the aerosol in the collection chamber to collect SARS‐CoV‐2 on the membrane. Then, the membrane filter with extracted SARS‐CoV‐2 is loaded in the injection chamber of the microfluidic chip based on the centrifugal fluid driving strategy and then records the fluorescence signals (Figure 3A). This system shows high efficiency in testing 115 clinical samples. Perarnau Ollé et al. [116] studied the conditions of enhancing the 3D‐printed microfluidic gas detectors for different VOCs of indoor air quality inside vehicles. In his work, a microfluidic chip made from poly‐methyl methacrylate (PMMA) and coated with polydimethylsiloxane (PDMS), which offers a good permeability to gases with high absorption properties, is then connected to the 3D‐printed housing that contained kit evaluation for VOCs to enhance the selectivity by controlling the conditions of the environments (Figure 3B). The sensor was used mainly for four types of VOCs that can be present in the indoor air of vehicles (ethanol, methanol, ethyl acetate, and toluene). It shows the efficiency in semi‐differentiating between the types. Still, it can be improved by a future study of microfluidic chips with multi‐channels coated with different polymers to increase the selectivity types. Ji et al. [117] developed a method of detecting pH aerosol online using a surface‐enhanced Raman spectroscopy (SERS) sensor and integrated it with a 3D‐printed microfluidic device (Figure 3C). As pH air aerosol, monitoring accuracy is very important because it affects the climate and atmospheric reactions and helps in understanding the mitigation of haze pollution. The integration of microfluidic devices represents the advantages of enhancing sensitivity and accuracy over a wide range of pH between 5 and 11 with a strong linear correlation (*R* ^2^ = 0.9727). As well, this integration also affords real‐time detection due to the controlled environment of the direct interaction of aerosol samples introduced to the side and reacted with modified substrate; then the Raman spectrometer laser probe will capture the SERS spectra related to variation in aerosol pH level. Furthermore, Yang et al. [118] used a microfluidic chip as part of the fabrication of an Array‐SERS chip to increase the sensitivity detection of multiplex gases like aldehydes, which are considered one of the toxic gas pollutants that lead to harmful diseases (Figure 3D). This combination of technologies helps increase molecular adsorption due to the control of gas flow and low background interferences, which led to a lower detection limit of 1 ppb compared to the traditional methods. Both studies show the efficiency of the integration of microfluidic technology with SERS sensors in increasing the sensitivity of the application sensors.

Microfluidic technology demonstrated a sophisticated manner of sensitivity and accuracy in sensing. On the other hand, several research studies reveal new horizons to presenting the advantages of this technology in different ways by repurposing the recreated human organs and tissue in small chips known as OoCs to assess the toxicity of air pollutants. For example, Shah et al. [119] discussed the importance of this kind of microfluidic, which is considered an artificial system to mimic human physiological and functionality conditions. One of the recreated organs is a lung‐on‐chip with the overview of different studies conducted on the effect of PM focusing on PM_2.5_ type that induced infections and diseases in pulmonary. The lung is considered a primary organ that is easily impacted as it directly reacts with air pollutants where they are deposited [120]. All the studies show good roots of applying the microfluidic devices technology in sensing and assessing air quality, which is a mandatory action required to be taken to adopt new technology to keep up with rapid change in life as the air can contain everything that can be lifted from the land with increasing population, urbanization, and climate change.

## 6 Summary

Despite its invisibility, the air can carry complex and dangerous substances that are challenging to investigate but crucial to understand. Table 3 summarizes key bioaerosols and allergens, including their mechanisms, seasonality, and clinical relevance. In the 18th century, urban pollution was primarily caused by horse‐drawn transport, with horse manure accumulating on streets, creating health risks due to allergenic dust from biological matter. Today, fuel‐powered engines have replaced horses, but PM emissions from vehicles are still linked to serious health effects, including increased cancer risk. Despite regulations mandating diesel particulate filters and manufacturer claims of “clean” cars, real‐world emissions often exceed lab standards. As a result, many cities are beginning to restrict or completely ban diesel vehicles from city centers. The growing availability of hybrid and fully electric vehicles, along with the expansion of supporting infrastructure, gives hope for a cleaner future [121, 122].

**TABLE 3:** Overview of major bioaerosols/allergens, their mechanisms, timing, and clinical significance.

Bioaerosol/Allergen	Examples	Pathophysiological effects	Seasonality	Role in respiratory diseases	Refs.
Fungal spores	*Aspergillus*, *Cladosporium*, *Alternaria*	Allergic rhinitis, hypersensitivity pneumonitis, asthma exacerbations; invasive infections in immunocompromised	Peaks late summer–autumn; indoor molds persist year‐round	Allergic fungal rhinosinusitis; severe asthma with fungal sensitization; hypersensitivity pneumonitis	[125]
Pollen	Grass, ragweed, birch	IgE‐mediated hypersensitivity; triggers allergic rhinitis, can exacerbate asthma	Seasonal: trees (spring), grasses (late spring–summer)	Major cause of seasonal allergic rhinitis and asthma exacerbations	[125]
Animal dander (cats, dogs)	Fel d 1, Can f 1–7	Allergenic proteins (e.g., Fel d 1, Can f 2/4/6) elicit IgE‐mediated airway inflammation; polysensitization associated with more severe asthma	Perennial; indoor exposure year‐round	Important triggers of perennial allergic rhinitis and asthma exacerbations; levels and specific components (e.g., lipocalins) correlate with asthma severity and morbidity	[125]
House dust mites	*Dermatophagoides pteronyssinus*, *D. farina*	Epithelial disruption; IgE‐mediated sensitization	Perennial; levels vary with humidity and temperature	Persistent allergic rhinitis and chronic asthma	[126]

Beyond transportation, industrial activities also contribute significantly to PM emissions. A global shift away from coal and diesel as energy sources is expected, reducing anthropogenic PM sources. With the right policies, human‐driven pollution can be mitigated. Natural sources, like dust storms, remain largely uncontrollable, but widespread deployment of monitoring stations can help in forecasting and tracking such events. Technological advances now enable PM detection and tracking not only via ground‐based stations but also through satellites, which can monitor particle size, chemical composition, and atmospheric reactions over vast regions [129]. With integrated satellite and ground‐level data, we can gain more accurate insights into pollutant types and their health impacts, enabling informed decisions about outdoor activity and personal protection. This approach is already implemented in several Asian cities, where citizens receive real‐time air quality updates via television, internet, and smartphones. Table 4 presents a comparative summary of international research on aerosols and health.

**TABLE 4:** Comparative overview of global studies on aerosols and health.

Region/Country	Aerosol(s)/Source	Health outcomes	Headline finding	Ref.
210 locations in 16 countries (global)	PM_2.5_ components (dust, nitrate, sulfate, crustal, sea salt)	All‐cause mortality	Mortality risk varies by chemical composition (higher for nitrate/OM in pooled models)	[129]
UAE (national, indoor)	Indoor PM_2.5_/PM_10_ and gases	Respiratory and neurologic symptoms	Higher indoor PM associated with more symptoms in adults and children	[129]
Africa (overview)	Household and ambient PM	Mortality	In 2019, air pollution was the second leading cause of death in Africa	[130]
Europe (32 countries; 654 regions)	Wildfire smoke PM_2.5_	Daily mortality	Smoke‐affected days linked to elevated short‐term mortality	[131]
The USA (national cohort)	Long‐term PM_2.5_ and components	All‐cause mortality	Higher long‐term PM_2_._5_ exposure linked to increased mortality	[132]

Various modeling tools exist for predicting the distribution and concentration of primary and secondary pollutants. These models, such as Pollution Climate Mapping (PCM) and ADMS‐Urban, help assess PM levels in areas lacking monitoring stations and project future pollution trends. Ultimately, early detection, effective policies, and robust monitoring technologies are key to minimizing air pollution. Long‐term data collection remains crucial for guiding decisions and improving public health outcomes.

Advances in OoC and microfluidic technologies enable long‐term monitoring of mammalian cell responses to pollutants, allowing toxicity assessment and dose determination without using animal models. Microfluidic systems can function as LOC for pollutant detection or OoC for simulating human organ responses to prolonged contaminant exposure. These technologies integrate with other methods to enhance testing efficiency. Air pollution, comprising particles, viruses, and gases such as VOCs, remains suspended in the atmosphere, posing risks to environmental quality and human health.

## Author Contributions

**Samar Damiati**: conceptualization, writing – original draft, investigation, validation, supervision. **Buthaina A. AlMashrea**: writing – original draft, investigation. **Nima Rabiei**: conceptualization, writing – original draft, methodology, validation. **Anju Prakasan Sujatha**: writing – original draft. **Dana Khdr Sabir**: writing – review and editing. **Mohamad Alhosani**: writing – review and editing. **Rimantas Kodzius**: conceptualization, investigation, writing – original draft, supervision, validation.

## Ethics Statement

The authors have nothing to report.

## Conflicts of Interest

The authors declare no conflicts of interest.

## Data Availability

Data sharing is not applicable to this article as no datasets were generated or analyzed during the current study.
